# Several Human Liver Cell Expressed Apolipoproteins Complement HCV Virus Production with Varying Efficacy Conferring Differential Specific Infectivity to Released Viruses

**DOI:** 10.1371/journal.pone.0134529

**Published:** 2015-07-30

**Authors:** Kathrin Hueging, Romy Weller, Mandy Doepke, Gabrielle Vieyres, Daniel Todt, Benno Wölk, Florian W. R. Vondran, Robert Geffers, Chris Lauber, Lars Kaderali, François Penin, Thomas Pietschmann

**Affiliations:** 1 Institute of Experimental Virology, TWINCORE, Centre for Experimental and Clinical Infection Research, Hannover, Germany; 2 Institute of Virology, Hannover Medical School, Hannover, Germany; 3 Department of General, Visceral and Transplant Surgery, Hannover Medical School, Hannover, Germany; 4 Integrated Research and Treatment Center Transplantation (IFB-Tx), Hannover Medical School, Hannover, Germany; 5 German Centre for Infection Research (DZIF), Partner Site Hannover-Braunschweig, Hannover, Germany; 6 Research Group Genome Analytics, Helmholtz Centre for Infection Research, Braunschweig, Germany; 7 Institute for Medical Informatics and Biometry, Medical Faculty, Technische Universität Dresden, Dresden, Germany; 8 Institut de Biologie et Chimie des Protéines, Bases Moléculaires et Structurales des Systèmes Infectieux, UMR 5086, CNRS, Labex Ecofect, University of Lyon, Lyon, France; Deutsches Primatenzentrum GmbH—Leibniz-Institut fur Primatenforschung, GERMANY

## Abstract

Apolipoprotein E (ApoE), an exchangeable apolipoprotein, is necessary for production of infectious Hepatitis C virus (HCV) particles. However, ApoE is not the only liver-expressed apolipoprotein and the role of other apolipoproteins for production of infectious HCV progeny is incompletely defined. Therefore, we quantified mRNA expression of human apolipoproteins in primary human hepatocytes. Subsequently, cDNAs encoding apolipoproteins were expressed in 293T/miR-122 cells to explore if they complement HCV virus production in cells that are non-permissive due to limiting endogenous levels of human apolipoproteins. Primary human hepatocytes expressed high mRNA levels of ApoA1, A2, C1, C3, E, and H. ApoA4, A5, B, D, F, J, L1, L2, L3, L4, L6, M, and O were expressed at intermediate levels, and C2, C4, and L5 were not detected. All members of the ApoA and ApoC family of lipoproteins complemented HCV virus production in HCV transfected 293T/miR-122 cells, albeit with significantly lower efficacy compared with ApoE. In contrast, ApoD expression did not support production of infectious HCV. Specific infectivity of released particles complemented with ApoA family members was significantly lower compared with ApoE. Moreover, the ratio of extracellular to intracellular infectious virus was significantly higher for ApoE compared to ApoA2 and ApoC3. Since apolipoproteins complementing HCV virus production share amphipathic alpha helices as common structural features we altered the two alpha helices of ApoC1. Helix breaking mutations in both ApoC1 helices impaired virus assembly highlighting a critical role of alpha helices in apolipoproteins supporting HCV assembly. In summary, various liver expressed apolipoproteins with amphipathic alpha helices complement HCV virus production in human non liver cells. Differences in the efficiency of virus assembly, the specific infectivity of released particles, and the ratio between extracellular and intracellular infectivity point to distinct characteristics of these apolipoproteins that influence HCV assembly and cell entry. This will guide future research to precisely pinpoint how apolipoproteins function during virus assembly and cell entry.

## Introduction

Recent estimates indicate that 80 million people worldwide are chronically infected with Hepatitis C virus (HCV), which is causing chronic liver disease leading to life-threatening conditions like cirrhosis and hepatocellular carcinoma [1]. While for many years, the standard anti-HCV therapy consisted of a combination of pegylated interferon-alpha and ribavirin, the first directly acting antivirals have recently been included in the therapeutic regimen. This has significantly improved treatment outcome. However, the high cost of these drugs limits their availability, especially in low income countries, and as a protective vaccine is not available, it will be difficult to rapidly reduce the global burden of HCV associated morbidity [2].

HCV particles circulating in the blood feature an unusually low and variable buoyant density, caused by the association of HCV with lipoproteins [3–5]. Lipoproteins are aggregates of lipids and (apolipo)proteins and are mainly produced in the liver and to a lower extent in the intestine. Lipoprotein association of HCV particles has several functions, including shielding of the virion from neutralizing antibodies, thereby contributing to the establishment of chronic infection [6]. Additionally, lipoproteins influence virus attachment and interaction of HCV particles with certain receptors like the low-density-lipoprotein-receptor (LDLR) and Scavenger receptor class B member 1 (SCARB1) on their target cells during entry [7]. Moreover, virus-resident ApoE is thought to facilitate particle attachment through binding to cell surface heparan sulfate [8]. Finally, several cellular factors involved in production of very low density lipoproteins (VLDL) like a ApoB, ApoE, and microsomal triglyceride transfer protein (MTTP) have been implicated in HCV particle production [9–13]. However, others and we have reported that ectopic expression of ApoE alone is sufficient to restore production of infectious HCV progeny in human non-liver cell lines, likely by interaction of ApoE with the viral glycoproteins during or after capsid envelopment [14–17]. Thus, ApoE expression seems to be the minimal cell-type specific requirement for production of infectious HCV particles. Of note, several liver cell expressed apolipoproteins, including ApoB and the exchangeable apolipoproteins E, A1, and C1 have been identified on HCV particles suggesting that they could act during virus assembly and may influence cell entry [4, 18–21]. Interestingly, ApoE, A1, and C1 form a family of evolutionary and structurally related proteins, together with ApoA2, A4, A5, C2, C3, and C4 [22–24]. All these apolipoproteins are secreted proteins, but their functions are diverse. While ApoE is important for the transport of dietary and endogenous lipids to peripheral tissues (reviewed in [25]), ApoA1 essential for reverse cholesterol transport from peripheral tissues back to the liver [26]. The ApoCs are believed to modulate receptor interactions and influence enzymes involved in lipid metabolism[27]. The role of these other exchangeable apolipoproteins in the HCV replication cycle has not been explored. Here we show that all ApoE-related exchangeable apolipoproteins can redundantly take over the role of ApoE in HCV assembly in non-liver cells and that amphipathic alpha-helical repeats within these proteins are the key determinant for their function. Distinct differences in the assembly efficacy, specific infectivity of released viruses and the ratio between extracellular and intracellular infectious virus production indicate that specific properties of these apolipoproteins influence these steps of the HCV replication cycle.

## Results

### Diverse Apolipoproteins are expressed in primary human hepatocytes

**Fig 1:**
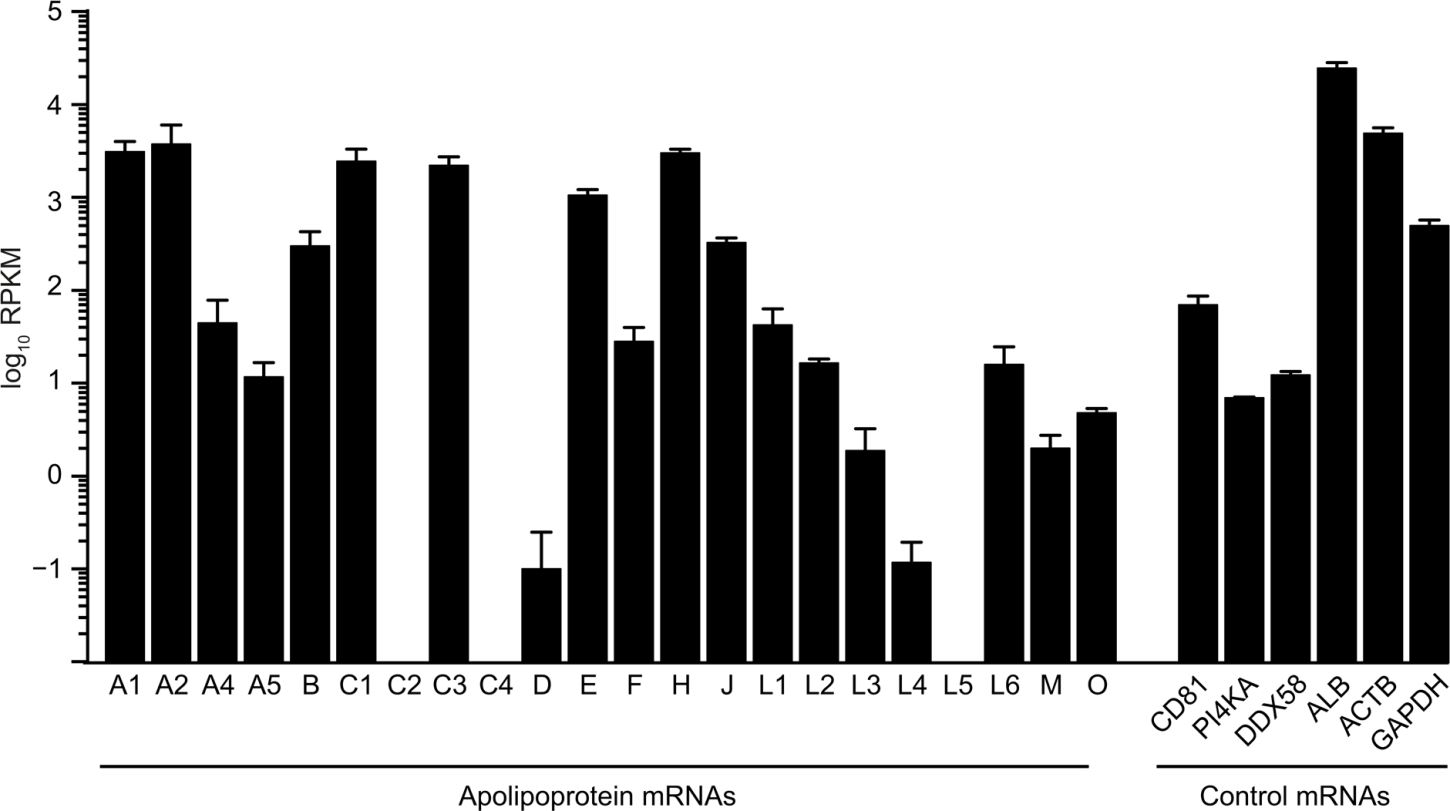
The mRNA of several exchangeable apolipoproteins is expressed in primary human hepatocytes. Average mRNA expression level of apolipoproteins are shown in primary human hepatocytes (PHH), compared to the HCV entry factor CD81, HCV host factor phosphatidylinositol 4-kinase alpha (PI4KA), innate immune sensor RIG-I (DDX58), the liver-specific host factor albumin (ALB), and two house-keeping genes ß-actin (ACTB), and GAPDH. The official gene ID for ApoJ is CLU (clusterin). Depicted are reads per kilobase per million reads (RPKM).

First, we examined mRNA expression levels of apolipoproteins in primary human hepatocytes (PHH), the predominant HCV target cells *in vivo*, by whole transcriptome RNA deep sequencing (Fig 1). As expected for liver cells, mRNA levels of ApoB and the exchangeable apolipoprotein ApoE were well detectable. Notably, among the ApoE-related exchangeable apolipoproteins, abundance of mRNAs coding for ApoA1, A2, C1, and C3 was even higher than the ApoE mRNA. Lower amounts were measured for ApoA4 and A5 while ApoC2- and C4-specific mRNA was absent. In addition, the mRNAs of many other unrelated exchangeable apolipoproteins were detectable (ApoD, F, H, J, L1, L2, L3, L4, L5, L6, M, O), albeit in part at low level. As a reference, we determined mRNA levels of crucial HCV host factors like the receptor CD81 [28] and the replication co-factor phosphatidylinositol 4-kinase III alpha (PI4KA) [29] and of DDX58 coding for the innate immunity sensor RIG-I (retinoic acid inducible gene I), which has been implicated in innate immune sensing of HCV infection [30]. Moreover, mRNA expression of the liver-specific host factor albumin (ALB) and of two house-keeping genes, β-actin (ACTB) and glyceraldehyde 3-phosphate dehydrogenase (GAPDH) was examined.

### ApoE-related exchangeable apolipoproteins can partially restore particle production in 293T/miR-122 cells

**Fig 2:**
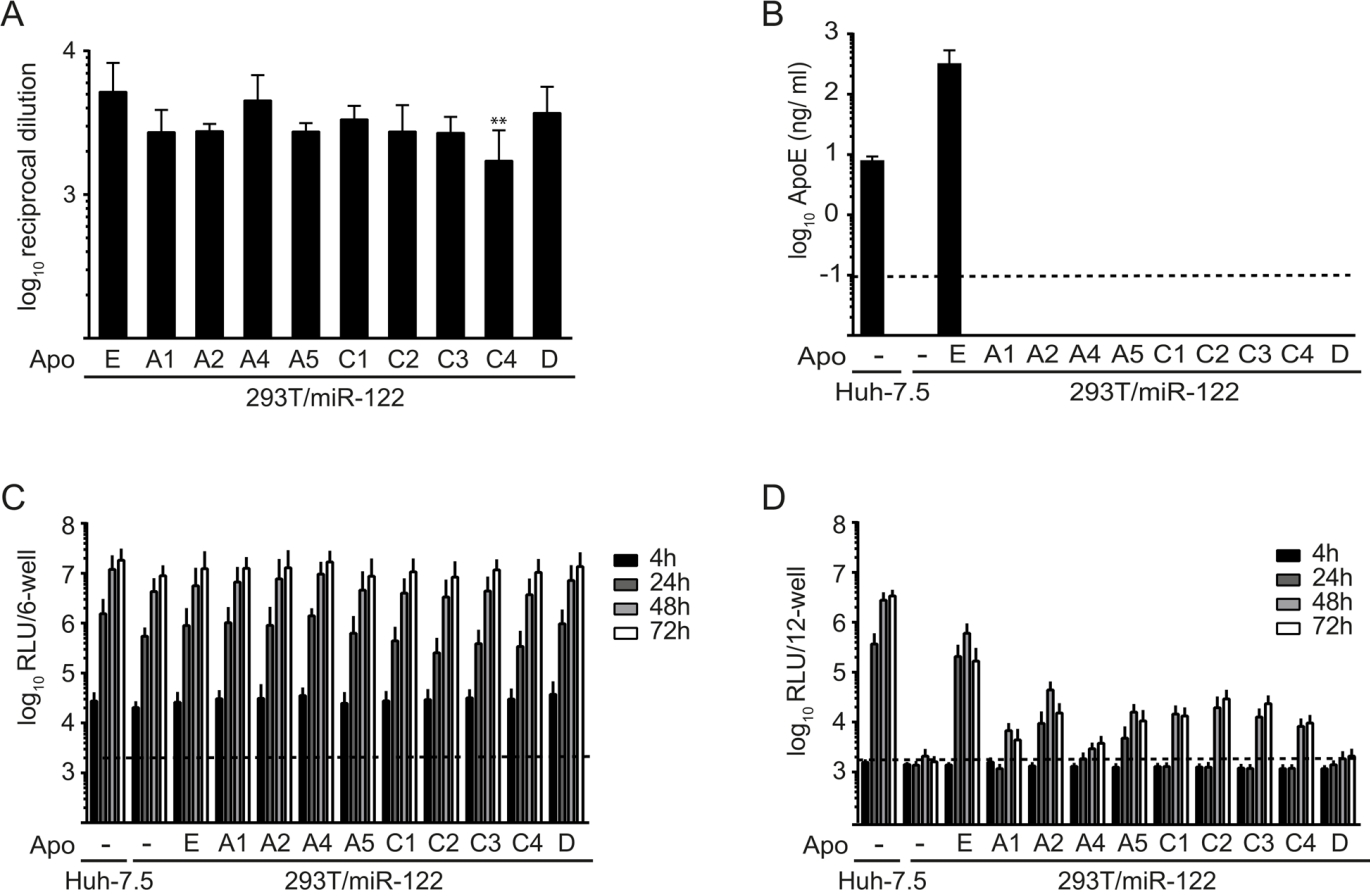
Ectopic expression of various exchangeable apolipoproteins in 293T/miR-122 cells restores production of infectious HCV particles. **A:** Depicted are reciprocal dilutions of secreted HA-tagged apolipoproteins that reach an OD of 2-fold over background (293T/miR-122 cells expressing an empty vector). Differences in secretion of individual apolipoproteins compared to ApoE were analyzed using one way ANOVA associated with Dunnett’s multicomparison test. Significant changes are indicated by asterisk (** = p < 0.01). **B:** Amounts of secreted ApoE (ng/ ml) in cell culture fluids were determined with a commercially available ELISA kit. The dashed line indicates the limit of detection. **C:** Cell lines were transfected with JcR2A HCV RNA encoding a *Renill*a luciferase reporter. HCV RNA replication was determined at 4 h, 24 h, 48 h and 72 h post transfection by luciferase measurements in the cell lysates and is expressed as relative light units (RLU). **D:** Cell-free culture fluids of transfected cells from (C) were used to inoculate naïve Huh-7.5 cells and infectivity was determined by luciferase assay at 72 h post inoculation. For panels (A), (B), (C), and (D) results are means and standard deviations (SD) of three independent experiments.

As the mRNA of many ApoE-related exchangeable apolipoproteins is highly abundant in primary human hepatocytes, we next explored the function of apolipoproteins in HCV morphogenesis. Given that HCV permissive human liver-derived cell lines like for instance Huh-7.5 cells express many apolipoproteins simultaneously, these cells cannot be used to dissect the contribution of individual apolipoproteins in HCV assembly. Therefore, we utilized human non-liver-derived 293T/miR122 cells, which robustly replicate HCV RNA but do not permit production of infectious progeny unless ApoE is ectopically expressed [14, 15]. To allow a quantitative comparison between expressed apolipoproteins we created C-terminally HA-tagged variants of all related exchangeable apolipoproteins (A1, A2, A4, A5, C1, C2, C3, C4, and E) as well as of ApoD (an unrelated exchangeable apolipoprotein) and introduced these into 293T/miR-122 cells via lentiviral gene transfer. Using an HA-tag specific ELISA assay we confirmed that all constructs are expressed and secreted at similar levels (Fig 2A). Moreover, we ruled out that expression of these apolipoproteins *trans*-activates secretion of ApoE using a commercially available ApoE-specific ELISA (Fig 2B). As expected, ApoE secretion was only observed in the cell culture fluid of Huh-7.5 cells and of 293T/miR-122 cells that had been transduced with ApoE-HA, but not in 293T/miR-122 cells that were transduced with the empty vector or with vectors encoding any of the other HA-tagged exchangeable apolipoproteins.

Subsequently, we determined the influence of individual apolipoproteins on the HCV replication cycle. Stable cell lines were transfected with GT2a-chimeric Jc1 reporter virus RNA expressing the *Renilla* luciferase gene and intracellular luciferase activity was monitored over time (Fig 2C). Accumulation of luciferase activity was comparable in all cell lines and was similar to Huh-7.5 cells, indicating that HCV replication was not modulated by expression of any HA-tagged apolipoprotein in 293T/miR-122 cells. Clarified supernatants were used to infect naïve Huh-7.5 cells, followed by assessment of luciferase activity at 72 h post infection (Fig 2D). As published previously, transduction of luciferase activity and thus presence of infectious HCV particles in the inoculum could be observed in cell culture fluids from Huh-7.5 cells and 293T/miR-122/ApoE cells, while no luciferase activity was transduced from the supernatant of 293T/miR-122 cells (Fig 2D and [15]). Importantly, all ApoE-related apolipoproteins rescued production of infectious HCV progeny in 293T/miR-122 cells, even though efficiency of luciferase transduction was reduced compared to cells expressing ApoE. Notably, the unrelated exchangeable apolipoprotein D did not enable detectable production of infectious particles in 293T/miR-122 cells, even though viral RNA replication was not influenced (Fig 2C and 2D).

**Fig 3:**
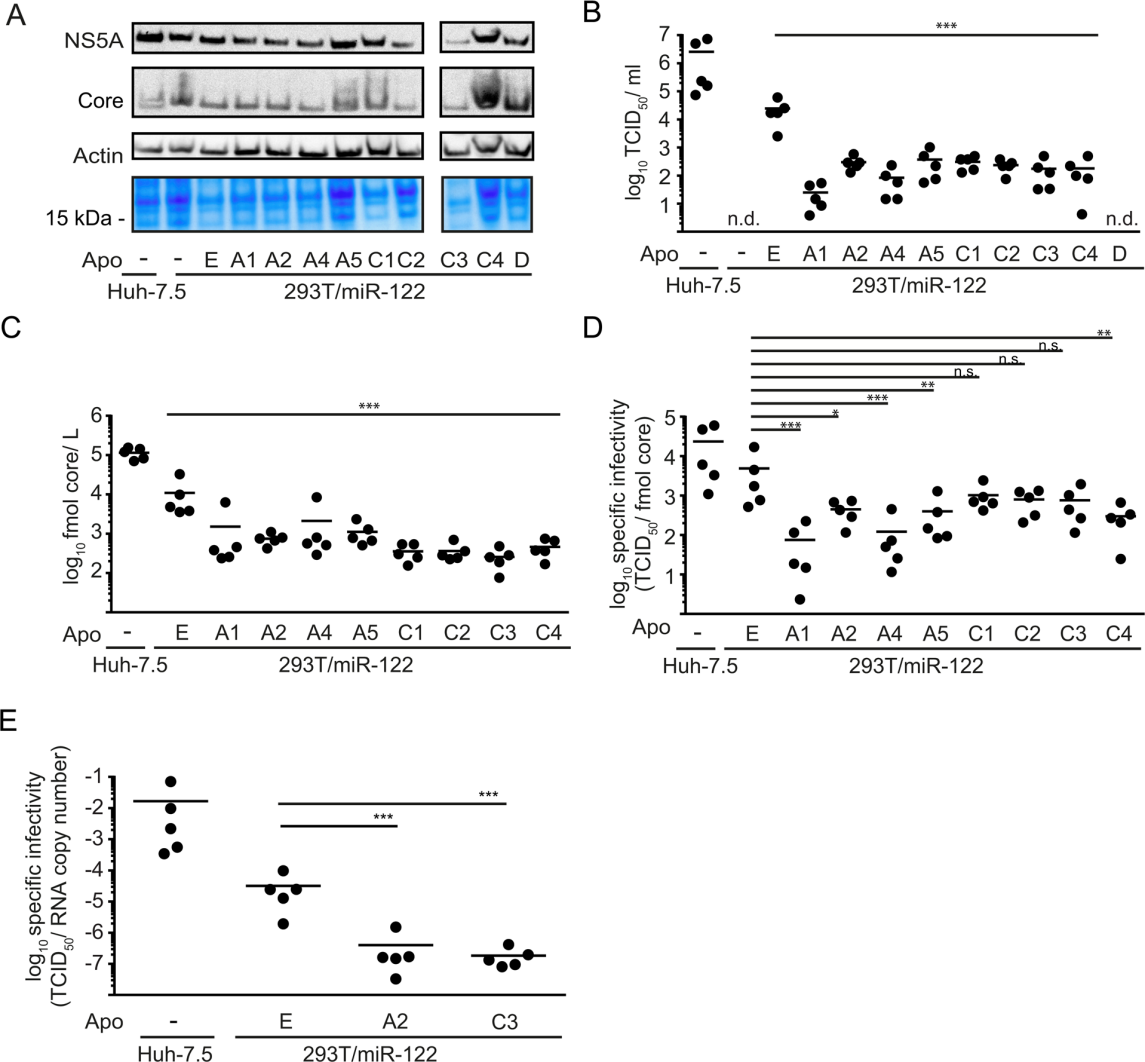
Expression of ApoA and ApoC protein family members complements HCV morphogenesis with reduced efficiency compared to ApoE. **A:** HCV RNA (Jc1 wt) was used to transfect indicated cell lines. Expression of viral and cellular proteins was verified by Western blot using antibodies against core, NS5A and β-actin. Additionally, total protein content was visualized by Coomassie staining. **B:** To assess production of infectious virus particles, supernatants were used to infect naïve Huh-7.5 cells to determine the titer by endpoint dilution assay (TCID_50_/ ml). **C:** Secretion of core protein into the cell culture supernatant was quantified by core-specific ELISA. Results are shown as fmol core/ L **D:** Specific infectivity of HCV particles released from the indicated cell lines was calculated based on the data presented in panel (B) and (C) and is given as TCID_50_/ fmol core. **E**: Specific infectivity was additionally calculated for a selection of cell lines based on RNA copy number released into the supernatant and corresponding TCID_50_ values. For (A), (B), (C), (D) and (E), all samples were taken 48 h post transfection and each data point represents an individual experiment while the mean is depicted as a dash. Statistical analyses in these panels were performed by Dunnett’s multicomparison test. Significant changes in infectivity, core secretion or specific infectivity of cells expressing other apolipoproteins compared to ApoE are indicated (*** = p < 0.001, ** = p < 0.01, * = p < 0.05). n. s. = non-significant (p > 0.05), n. d. = not detected

To corroborate these results, we transfected the cell lines with parental Jc1 wild-type RNA lacking a luciferase reporter gene. Similar expression levels of viral and cellular proteins were verified via Western blot with specific antibodies against the viral core and NS5A proteins and against cellular β-actin as well as by Coomassie staining of total protein content (Fig 3A). To assess production of virus particles, clarified supernatants obtained 48 h post transfection were used to infect Huh-7.5 cells to determine the virus titer via end point dilution assay (TCID_50_) (Fig 3B). Similar to what we observed with the HCV reporter construct, expression of ApoD did not restore detectable production of infectious HCV particles in this very sensitive assay. However, again all ApoE-related exchangeable apolipoproteins were able to sustain particle production, though to a significantly lower extent compared to ApoE. Thus, all ApoE-related exchangeable apolipoproteins of the ApoA and ApoC family could at least partially take over the role of ApoE in HCV morphogenesis.

To elucidate why virus infectivity was reduced in cells expressing ApoE-related proteins, secretion of core protein, indicative of particle release, was quantified by ELISA (Fig 3C). ApoE related apolipoproteins were significantly less efficient in supporting core protein secretion than ApoE itself. As HCV RNA replication and expression levels of intracellular core and NS5A protein were not influenced by apolipoprotein expression (see Figs 2C and 3A), we concluded that exchangeable apolipoproteins other than ApoE sustain particle assembly and/ or secretion with lower efficiency in 293T/miR-122 cells. Additionally, we calculated the specific infectivity of particles produced in the presence of the individual apolipoproteins, based on data shown in Fig 3B and 3C (Fig 3D). As published before, particles produced in 293T/miR-122/ApoE cells featured a lower specific infectivity compared to Huh-7.5-derived particles, indicating that other important host factors that have so far not been identified were lacking in 293T/miR-122/ApoE cells [15]. Specific infectivity of particles produced in 293T/miR-122 in the presence of ApoC1, C2 and C3 was similar to values observed from ApoE-expressing cells. In contrast, a significant reduction could be detected for ApoA1, A2, A4, A5 and C4, indicating that not only less physical HCV particles were produced in these cells but that these particles were also less infectious (Fig 3D). To corroborate these data, we quantified the specific infectivity of HCV particles produced in a set of cell lines based on TCID_50_ and HCV genome copies (Fig 3E). Again, a significantly reduced specific infectivity of particles produced in 293T/miR-122/ApoA2 compared to ApoE was evident. Notably, in this set of experiments, particles produced in the presence of ApoC3 also had a significantly lower specific infectivity compared to those produced with ApoE. Taken together, these observations highlight the unique function of ApoE during assembly and cell entry which cannot be fully compensated by other ApoE-related apolipoproteins.

**Fig 4:**
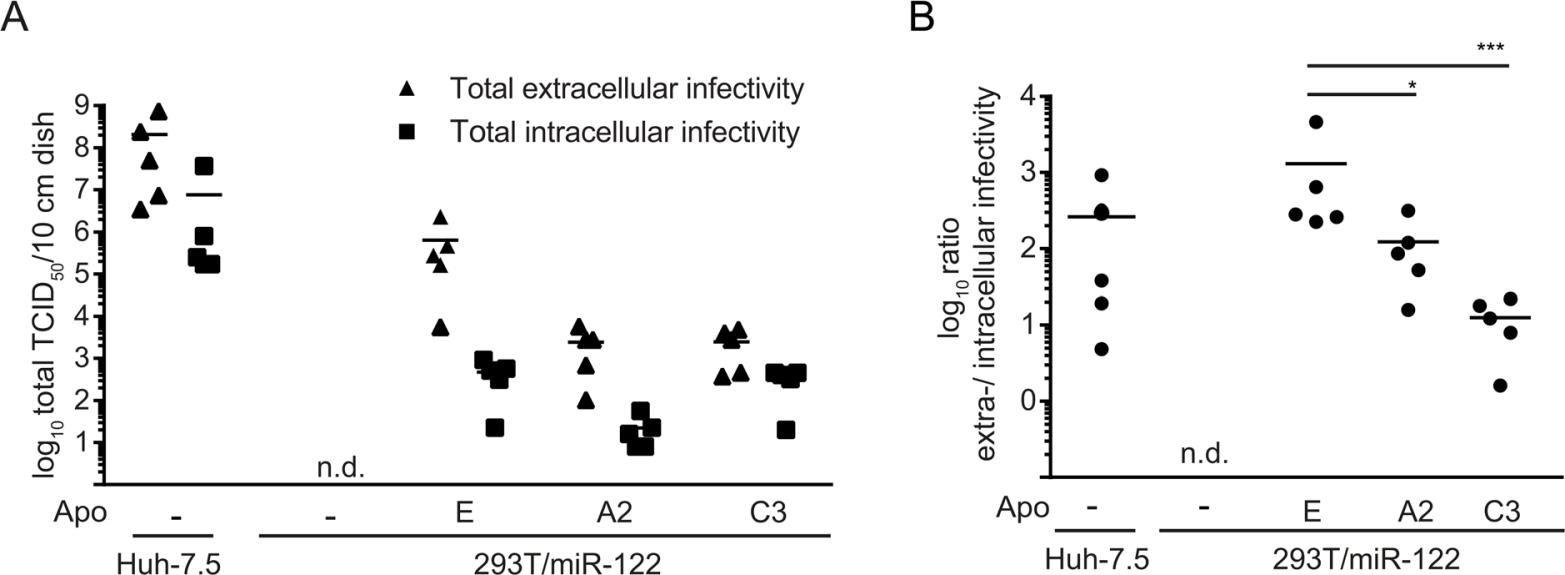
Different ratio between extracellular and intracellular infectious virus production in HCV transfected Huh-7.5 and 293T/miR-122 cells. **A:** Depicted cell lines were transfected with HCV RNA (Jc1) and after 48 h, production of infectious virus particles was assessed in the cell culture supernatants as well as in freeze-thaw lysates by limited dilution assay. Shown are the viral titers per 10 cm culture dish. **B:** Ratio of extra- to intracellular infectivity was calculated based on data presented in panel (A). Statistical analyses were performed by Dunnett’s multicomparison test. Significant changes in infectivity of cells expressing ApoA2 or ApoC3 compared to ApoE are indicated. (* = p < 0.005, *** = p<0.001).

To further investigate the impact of individual apolipoproteins on different steps of HCV morphogenesis, we compared production of intracellular and extracellular infectivity for selected cell lines by quantifying HCV infectivity in the culture fluid and in freeze and thaw extracts of transfected cells. As expected, 293T/miR-122 cells expressing ApoA2 or ApoC3 released ca. 100-fold lower levels of infectious HCV compared to ApoE expressing 293T/miR-122 cells (Fig 3C and Fig 4A). In contrast, production of intracellular infectious particles was comparable between ApoE and ApoC3 expressing 293T/miR-122 cells and ca. 10-fold lower in case of 293T/miR-122 ApoA2 cells (Fig 4A). Consequently, the ratio between extracellular and intracellular infectious HCV particles was significantly reduced in 293T/miR-122 cells expressing ApoA2 or ApoC3 compared to ApoE (Fig 4B).

### Amphipathic alpha-helical repeats within ApoC1 are the key determinant for its function in HCV particle production

**Fig 5:**
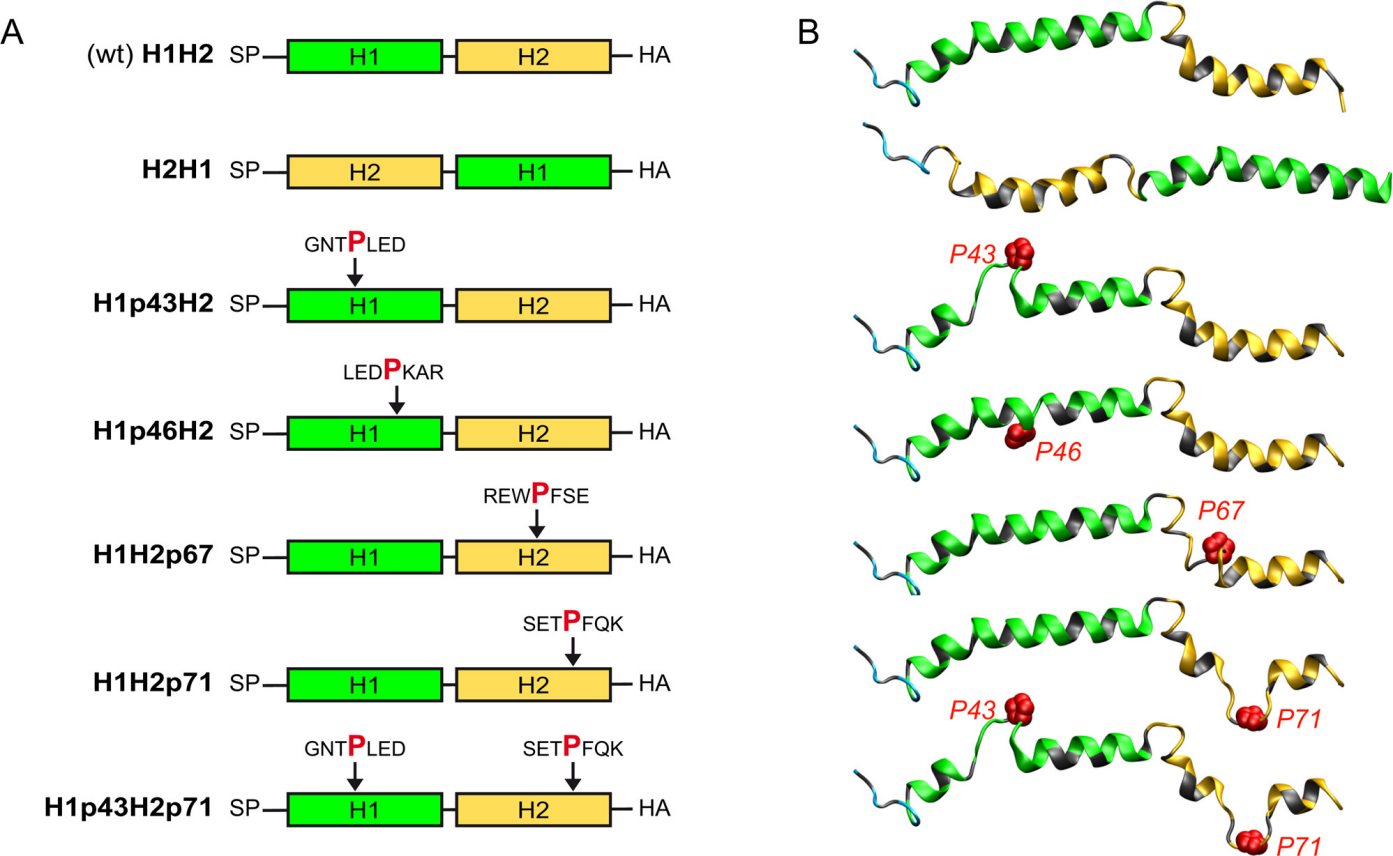
Schematic model and ribbon representation of wild type ApoC1 and mutant proteins. **A:** Schematic depiction and **B:** predicted 3D structural models of wild-type ApoC1 (H1H2) and six mutants including swapped helices (H2H1), and proline insertions after given amino acid positions to disrupt formation of the first helix (H1p43H2 and H1p46H2) or the second helix (H1H2p67 and H1H2p71), or both helices (H1p43H2p71). Hydrophobic residues are colored grey while polar residues are colored cyan, green and yellow in the N-terminus segment, helix 1 and helix 2, respectively. The side-chain atoms of inserted proline residues are represented as red spheres of the corresponding van der Waals radius. The reader is looking at the polar sides of helices 1 and 2 (green and yellow, respectively) while their hydrophobic sides (colored gray) are on the opposite side, interacting with the membrane surface (represented by the background). SP = signal peptide

**Fig 6:**
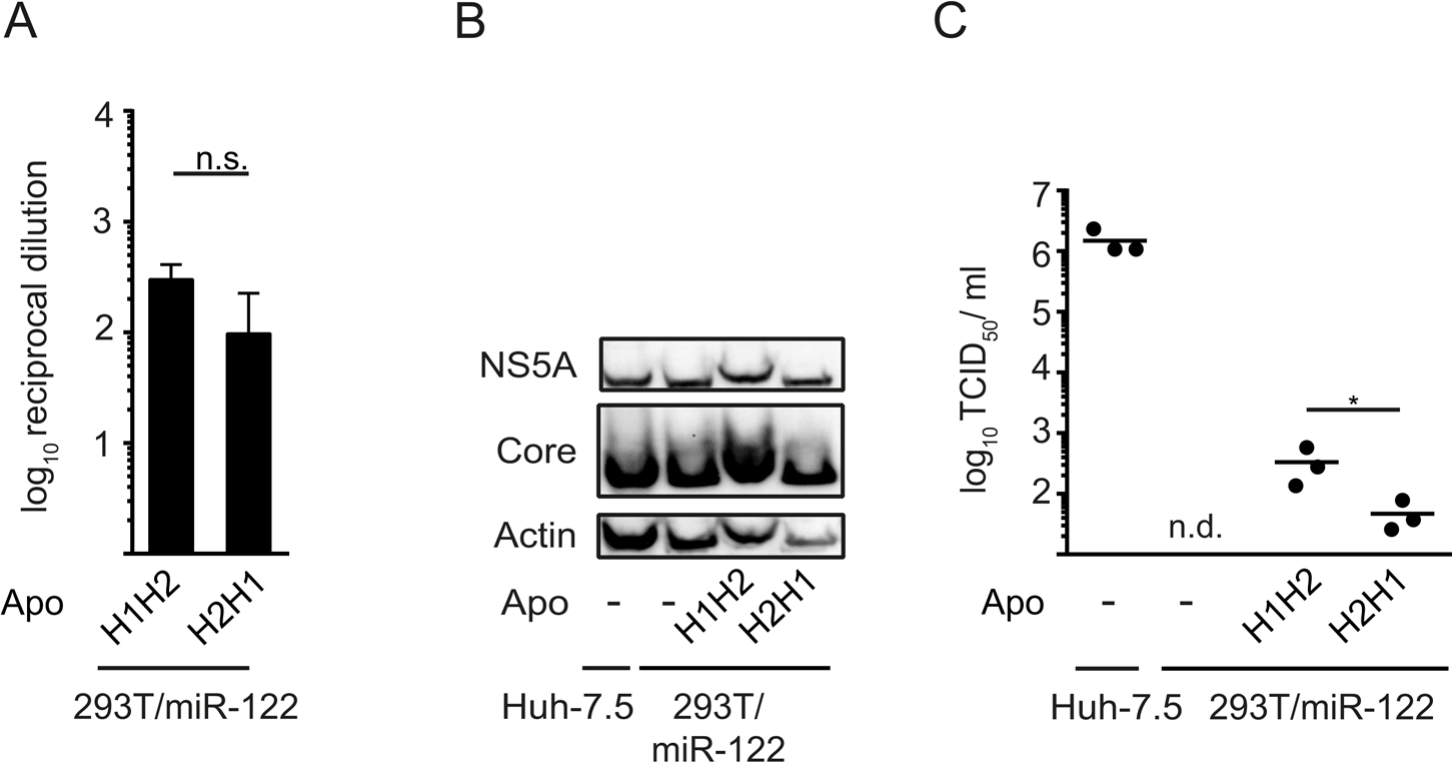
Switching the order of amphipathic alpha-helices in ApoC1 does not ablate its function in production of infectious HCV particles. **A:** Secretion of wild-type ApoC1 (H1H2) and a mutant containing swapped amphipathic alpha-helices (H2H1) was quantified via HA-specific ELISA. Depicted are average values of 3 independent experiments with reciprocal dilutions of secreted proteins that reach an OD of 2-fold over background (cells expressing the empty vector). Significant changes compared to wild-type ApoC1 were calculated based on one-tailed unpaired t test with Welch’s correction. n.s. = non-significant (p > 0.05). **B:** and **C:** Depicted cell lines were transfected with HCV RNA (Jc1 wt) by electroporation and the expression of core protein, NS5A and ß-actin were determined by Western blotting. **C:** Absolute values of viral infectivity were determined by end point dilution assay. Plotted are individual results of three independent experiments with means presented as a dash. Data were analyzed by one-tailed unpaired t test with Welch’s correction (* = p < 0.05), n. d. = not detected.

Given that all ApoE-related apolipoproteins at least partially restored particle production from 293T/miR-122 cells, we hypothesized that these proteins should share a common determinant for their function in HCV morphogenesis. Amphipathic alpha-helical repeats are present in all ApoE-related exchangeable apolipoproteins but are absent in unrelated apolipoproteins like ApoD and ApoH [24, 31–33]. However, even though this structural feature is conserved, the number of helices as well as their primary amino acid sequence varies considerably between family members. To define if these helices constitute the minimal requirement for production of infectious HCV, we focused on ApoC1 for further analyses due to its small size. NMR studies revealed that mature ApoC1 essentially is a bi-helical protein in which two helices are connected by a short linker (Fig 5A and 5B) [34]. Initially, we created a HA-tagged variant of ApoC1 in which both helices were left intact, but the order of helices was switched (H2H1), according to a previous publication [35]. We transduced 293T/miR-122 cells with this construct and excluded a significant impact of this mutation on its secretion by HA-specific ELISA (Fig 6A). Next, we determined, if viral protein expression was affected after transfection with HCV RNA. As we did not observe major differences in abundance of NS5A or core protein between cells expressing the wild-type (H1H2) or mutant ApoC1 (H2H1) (Fig 6B), we next assessed particle production. Remarkably, even though the sequence of helices in ApoC1 was switched, this protein still enabled particle production from 293T/mir-122 cells, albeit with slightly reduced efficiency compared to parental ApoC1 (Fig 6C).

**Fig 7:**
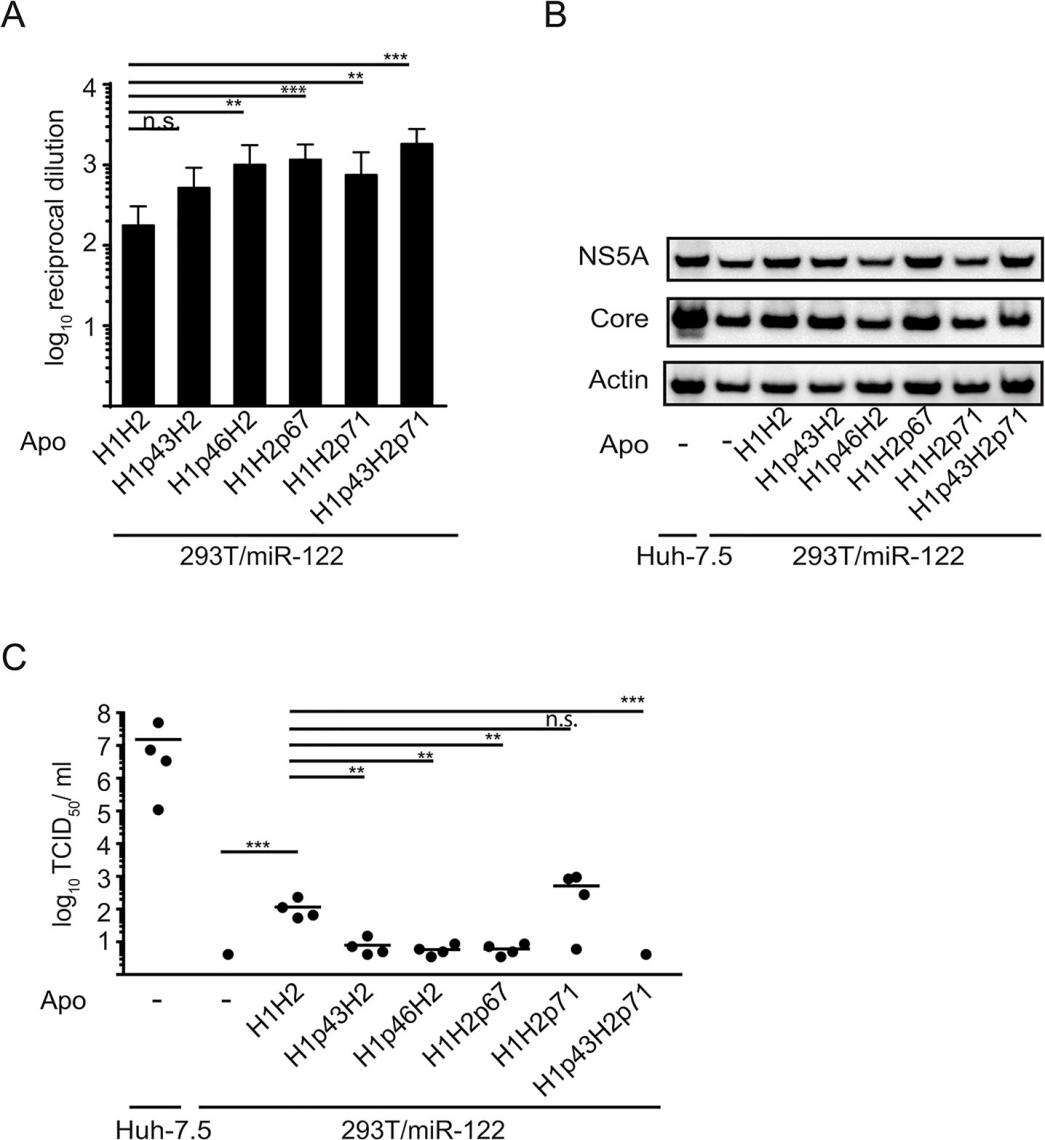
Amphipathic alpha-helices in ApoC1 are key determinants for HCV assembly. **A:** Secretion wild-type ApoC1 (H1H2) and five mutants with proline insertions to disrupt formation of the first helix (H1p43H2 and H1p46H2) or the second helix (H1H2p67 and H1H2p71), or both helices (H1p43H2p71) were quantified by HA-specific ELISA. Depicted are average values of 3 independent experiments with reciprocal dilutions of ApoC1 constructs that reach an OD of 2-fold over background. Significant changes compared to wild-type ApoC1 are indicated based on Dunnetts’ multicomparison test. (*** = p < 0.01, ** = p < 0.01, n.s. = non-significant (p > 0.05)) **B:** Cells transfected with HCV RNA (Jc1 wt) and expression of viral and cellular proteins was determined by Western blotting using antibodies specific for core, NS5A and ß-actin. **C:** Absolute values of viral infectivity were determined by end point dilution assay. Plotted are individual results of four independent experiments with means presented as a dash. Data were analyzed by Dunnett’s multicomparison test. (*** = p < 0.01, ** = p < 0.01, n.s. = non-significant (p > 0.05))

To further define crucial protein determinants for HCV morphogenesis, we additionally mutated ApoC1 by proline insertions to disrupt helix formation. To this end a proline residue was inserted within helix 1 (at position 43 or 46) or helix 2 (at position 67 or 71) or simultaneously into both helices (at position 43 and 71) (see schematic overview Fig 5A). Fig 5B shows a model of the potential impact of proline insertions on helix formation. Analogous to our previous assays, we created stable 293T/miR-122-derivatives expressing these ApoC1 variants. Again, proline insertions did not reduce secretion of the ApoC1 variants, but even significantly increased secretion for most constructs as determined by ELISA (Fig 7A). Furthermore, as seen with the ApoC1 construct with switched helices, the different mutants did not influence HCV protein expression (Fig 7B). Yet, compared to parental ApoC1, proline mutations in helix 1 (H1p43H2 and H1p46H2) significantly impaired production of infectious particles (Fig 7C). For helix 2, only a proline insertion at position 67 reduced virus titer while a proline at position 71 did not show a significant effect compared to wild-type ApoC1. However, if an additional proline was inserted at position 43 in helix 1, thereby impairing both helices in ApoC1 simultaneously (H1p43H2p71), particle production was abrogated. In conclusion, these results indicate that amphipathic alpha-helices within ApoC1 are the key determinant for its role in production of infectious HCV particles.

## Discussion

It is well-known that during its replication cycle HCV closely interacts with lipoprotein metabolism, particularly with the VLDL pathway, as several components of this pathway like ApoB, ApoE and MTTP have been implicated in HCV morphogenesis [9–13]. However, previous studies could show that expression of ApoE alone is sufficient to restore production of infectious HCV progeny in non-liver cells, indicating that ApoB and MTTP are not essential [14, 15]. Nevertheless, these factors and other exchangeable apolipoproteins could modulate HCV assembly and particle properties. In this study, we demonstrate that all ApoE-related exchangeable apolipoproteins of the ApoA and ApoC families complement the lack of ApoE in 293T/miR-122 cells during HCV production. However, HCV assembly, the specific infectivity of released particles and the ratio between extracellular and intracellular infectious particles was significantly different between ApoE and members of the ApoA and ApoC lipoprotein families (Fig 3 and Fig 4). Therefore, ApoE has unique functions/properties during HCV morphogenesis and cell entry which are not fully complemented by these other lipoproteins. It has been shown that ApoE associated with HCV particles can influence viral attachment by way of interaction with cell surface heparin sulfate and proteins like SCARB1 and LDL-R which have been implicated in HCV entry [3, 8, 36–38]. While in addition to ApoE, at least ApoA1, A2, and C3 were also shown to interact with SCARB1, it is very well possible that subtle changes in affinity for this receptor may reduce entry efficiency and thus affect specific infectivity [39–43]. Future studies investigating these differences may reveal new details about how these host factors influence HCV infectivity. Moreover, since primary human hepatocytes abundantly express several apolipoproteins (ApoA1, A2, C1, C3, E) that are capable of sustaining HCV particle production, it is possible that HCV virions incorporate a mixture of these proteins which influences viral cell entry.

Interestingly, ApoD, which is unrelated to ApoE, did not restore particle production, indicative of conserved features of all ApoE-related apolipoproteins. Indeed, ApoE and all ApoA and ApoC family members contain domains of amphipathic alpha-helical repeats, while ApoD does not harbor these helices. By inserting proline residues to impair helix formation in ApoC1, we provide evidence that amphipathic alpha-helical repeats are the key structural determinant for particle production.

While this work was in progress, Fukuhara *et al*. published an elegant and comprehensive study reporting that ApoA1, A2, C1, C2, and C3 but not ApoH compensate for ApoE function in HCV assembly in 293T cells and in engineered Huh-7 cells in which ApoB and ApoE were knocked-out [44]. These results partially overlap with our observations. However, beyond this very recent study, we additionally show that ApoA4, A5, and C4 are functional in HCV assembly and cell entry. Moreover, Fukuhara *et al*. and our study provide independent confirmation for the conclusion that amphipathic helices are crucial for particle assembly. While they determined the importance of these helices by using deletion mutants of ApoE and ApoC1 in Huh7 double-knockout cells, we impaired helix formation by insertion of proline residues in ApoC1 and determined particle production in 293T/miR-122 cells. Thus, both studies highlight the flexible use of apolipoproteins during HCV assembly and infection and they underscore the critical role of amphipathic helices in these processes. In fact, we show that the sequence of helices in the context of ApoC1 is not absolutely essential for functioning in HCV assembly since swapping helix one and two did not abrogate virus production. It will be interesting to further explore by which mechanisms these amphipathic helices support HCV assembly and which features are critical during cell entry. Of note, Fukuhara *et al*. observed comparable virus production and specific infectivity between ApoA1, A2, C1, C2, C3, and E in Huh-7 double knock-out cells, whereas we observed significant differences between apolipoproteins to restore virus production in 293T/miR-122 cells. In addition, Fukuhara *et al*. also observed variable efficiency of HCV virus production by apolipoproteins expressed in 293T cells, although these differences were not fully consistent with our findings. These discrepancies may in part relate to varying experimental procedures. For instance, Fukuhara *et al*. used untagged variants of these apolipoproteins compared to HA-tagged variants in our study. As a consequence we were able to confirm comparable expression level between these apolipoproteins during our assays. Future studies based on these epitope tagged apolipoprotein variants may provide new insights into the composition of HCV particles that could explain differences in the specific infectivity and in the functioning of these proteins during HCV assembly and cell entry. Clarifying the molecular basis for these differences and using the models described by Fukuhara *et al*. and in this study should prove valuable to dissect additional details of the interplay between HCV and lipoproteins during virus assembly and infection.

## Materials and Methods

### Primary human hepatocytes

Primary human hepatocytes (PHH) were obtained from the department of General, Visceral, and Transplant Surgery at Hannover Medical School (Hannover, Germany) or from Pimacyt (Schwerin, Germany) and cultured as described elsewhere [45]. Human liver tissue for cell isolation was obtained from four patients undergoing partial hepatectomy and after written informed consent was obtained. The protocol was approved by the ethics commission of Hannover Medical School (Ethik-Kommission der MHH, #252–2008). For quantification of mRNA expression level, total RNA was isolated by Trizol extraction following manufacturer’s instructions (Invitrogen) and sequenced on an Illumina HiSeq 2000 platform.

### Cell culture

Huh-7.5 [46] and 293T/miR-122 derivatives were maintained in Dulbecco’s Modified Eagle Medium (Invitrogen) containing 10% fetal calf serum (PAA Laboratories GmbH), 2 mM L-glutamine (Invitrogen), 1 mM non-essential amino acids (Invitrogen), and 100 μg/ml penicillin/ streptomycin (Invitrogen). 293T/miR-122 cells [15] were transduced with lentiviruses as described previously [47] to ectopically express apolipoprotein constructs. If appropriate, 2 μg/ml puromycine (Sigma) or 5 μg/ml blasticidine (Invivogen) for selection of transgene-expressing cells were added.

### Plasmid constructs

The genotype 2A/2A chimera Jc1 [48], the *Renilla* luciferase reporter virus JcR2A[49], the pLenti plasmid harboring the miR-122 gene [50], and the human ApoE construct with a C-terminal HA-tag [51] have been described previously. Plasmid constructs harboring the various apolipoproteins ApoA1, A2, A4, A5, C1, C2, C3, C4, and D, as well as the ApoC1 variants were created by synthesizing DNA fragments at IDT technologies that were cloned into a pWPI plasmid by standard cloning strategies. All new constructs were verified by DNA sequencing at MWG Eurofins. Detailed cloning strategies are available upon request.

### Enzyme-linked immunosorbent assay (ELISA)

To quantify amounts of secreted apolipoproteins, stable cell lines ectopically expressing different apolipoproteins harboring C-terminal HA-tags were seeded at a density of 2 x 10^5^ cells per well of a 6-well plate and incubated for 48 h. To reduce protein content, medium was replaced with Adenovirus expression Medium (Invitrogen) after 36 h. Clarified supernatants were coated onto F96Maxisorp Nunc-Immuno plates (Thermo Scientific) over night at 4°C. Remaining protein binding sites were blocked with 5% bovine serum albumin (BSA, Gibco) in PBS followed by immuno staining with a primary mouse anti-HA antibody (1:1000; Covance) and a secondary anti-mouse antibody coupled to horseradish peroxidase (HRP) (1:20000; Sigma) for 1 h at room temperature. Washing steps were carried out with PBS with 0.05% (v/v) Tween-20 (Sigma). 3,3,5,5’ Tetramethylbenzidine (TMD; Sigma) was used as a substrate for the HRP-coupled antibody. The reaction was stopped by addition of 1 N sulfuric acid and enzyme activity was quantified 450 nm with an ELISA plate reader (BioTech). Reciprocal dilutions of secreted HA-tagged apolipoproteins that reach an OD of 2-fold over background (293T/miR-122 expressing the empty vector) were calculated based on these data.

Amounts of secreted ApoE in cell culture fluids were determined with a commercially available ELISA kit according to the manufacturer’s instructions (Mabtech).

To quantify core protein secretion, clarified supernatants were harvested 48 h post transfection and were inactivated with Triton X-100 (Roth) at a final concentration of 1% (v/v) and analyzed with a diagnostic kit (Architect Anti-HCV; Abbott).

### *In vitro* transcription and transfection

*In vitro* transcripts and RNA transfection by electroporation was performed as described by Steinmann *et al*. Transfected cells were immediately transferred into 16 ml of medium and 2 ml of cell suspension were seeded per well of a 6-well plate.

### Quantification of virus infectivity

Standard infection assays with *Renilla* luciferase reporter viruses were carried out as published [15].

For the Jc1 chimera, extracellular viral titers were determined via end point dilution assay (TCID_50_) as described previously [52]. Quantification of intracellular HCVcc was determined as described in Hueging *et al*. [15].

### Quantitative detection of viral RNA

Conditioned supernatants containing HCV RNA were isolated 48 h post transfection and viral RNA was isolated according to the manufacturer’s instructions (High Pure Viral RNA Kit; Roche). For quantification, a one-step RT-PCR Light-Cycler 480 RNA Master Hydrolyis Probe Kit (Roche) was employed as described earlier ([51])

### Immunoblotting

Expression of viral and cellular proteins was verified via Western blot as described elsewhere [15] using anti-core (C7-50; 1:1000 [53]) anti-NS5A (9E10; 1:1000 [54]) anti-ß actin-HRP (1:20000; Sigma) and anti-mouse-HRP (1:20000; Sigma) antibodies. Antigens were detected with the ECL prime detection reagent (GE Healthcare).

Protein expression was further visualized by Coomassie staining with 45% methanol, 10% acetic acid, and 0,1% Coomassie blue R250 for 10 minutes, followed by destaining with 45% methanol and 10% acetic acid overnight.

### Structural Models of ApoC1

NMR model of wild-type ApoC1 (top, PDB entry 1IOJ [34]) was used as template to construct the models of proline insertion mutants by using Swiss Model facilities (http://swissmodel.expasy.org) [55]. Figures were generated from structure coordinates using VMD (http://www.ks.uiuc.edu/Research/vmd/) and rendered with POV-Ray (http://www.povray.org/).
